# Extracranial anticoagulant related bleedings admitted to intensive care units: a French multicenter retrospective study

**DOI:** 10.1186/s13054-023-04605-4

**Published:** 2023-08-09

**Authors:** Thomas Botrel, Sibylle Cunat, Julie Helms, Jérémie Lemarié, Jeanne Gaillon, Sébastien Préau, Raphael Favory, Arnaud W. Thille, Florence Boissier, Eric Maury, Jérémie Joffre, Hafid Ait-Oufella

**Affiliations:** 1 Medical Intensive Care Unit, Intensive Care Department, Saint Antoine University Hospital, APHP, Sorbonne University, 75012 Paris, France; 2 Université de Strasbourg (UNISTRA), Faculté de Médecine, Hôpitaux Universitaires de Strasbourg, Service de Médecine Intensive-Réanimation, Nouvel Hôpital Civil, Strasbourg, France; 3 INSERM (French National Institute of Health and Medical Research), UMR 1260, Regenerative Nanomedicine (RNM), FMTS, Strasbourg, France; 4 Intensive Care Department, Nantes University Hospital, Nantes, France; 5 Intensive Care Department, CHU Lille, Univ. Lille, RID-AGE, INSERM UMR 1167, Institut Pasteur, 59000 Lille, France; 6 Intensive Care Department, Centre Hospitalo-Universitaire de Poitiers, Poitiers, France; 7 INSERM CIC 1402 (IS-ALIVE Group), Université de Poitiers, Poitiers, France; 8 Sorbonne Université, Paris, France; 9 Centre de Recherche Saint-Antoine Inserm UMR-S 938, Paris, France

**Keywords:** Anticoagulant, Bleeding, Transfusion, ICU, Outcome

## Abstract

**Background:**

Anticoagulants are widely used but can lead to iatrogenic events such as bleeding. Limited data exists regarding the characteristics and management of patients admitted to intensive care units (ICU) for severe anticoagulant-related extracranial bleeding.

**Methods:**

A retrospective observational study was conducted in five French ICUs. From January 2007 to December 2018, all patients aged over 18 years admitted to ICU for extracranial bleeding while receiving therapeutic anticoagulation were included.

**Results:**

486 patients were included, mainly male (61%) with an average age of 73 ± 13 years. Most patients had comorbidities, including hypertension (68%), heart disease (49%) and diabetes (33%). Patients were treated by vitamin K antagonists (VKA, 54%), heparins (25%) and direct oral anticoagulants (DOAC, 7%). The incidence of patients admitted to ICU for anticoagulant-related bleeding increased from 3.2/1000 admissions in 2007 to 5.8/1000 in 2018. This increase was particularly high for DOAC class. Upon admission, patients exhibited severe organ failure, as evidenced by a high SOFA score (7 ± 4) and requirement for organ support therapies such as vasopressors (31.5%) and invasive mechanical ventilation (34%). Adherence to guidelines for the specific treatment of anticoagulant-related bleeding was generally low. ICU mortality was 27%. In multivariate analysis, five factors were independently associated with mortality: chronic hypertension, need for vasopressors, impaired consciousness, hyperlactatemia and prolonged aPTT > 1.2.

**Conclusion:**

Anticoagulant-related extracranial bleeding requiring ICU admission is a serious complication responsible for organ failure and significant mortality. Its incidence is rising. The therapeutic management is suboptimal and could be improved by educational programs.

**Supplementary Information:**

The online version contains supplementary material available at 10.1186/s13054-023-04605-4.

## Introduction

Anticoagulants are among the most widely used medications worldwide [1]. In France, more than 3 million patients (5% of the population) received anticoagulant therapy in 2013 [2]. These medications are essential for preventing or treating arterial and venous thrombotic events and other widespread cardiovascular conditions associated with significant morbidity, mortality and economic consequences [3]. Heparin and its derivatives are usually administered during the acute phase of illness, while vitamin K antagonists (VKA), and more recently new molecules labeled “sdirect oral anticoagulants” (DOAC) are available for chronic treatment [2, 4]. However, anticoagulant medication is associated with a high rate of adverse events. In a large study in the United States, anticoagulants were identified as the primary medication responsible for adverse drug-related events requiring emergency hospitalizations in older adults [5]. Bleeding is the main life-threatening side effect of anticoagulant therapy, and varies according to the type of molecule [3]. Patients treated with DOACs experience lower bleeding rates compared to those receiving VKAs. For instance, Steinberg et al. reported a major bleeding incidence of 3.3 per 100 patient-years for patients treated by DOAC, compared to 3.5 per 100 patient-years when patients received VKA [6]. Similarly, in a multicenter cohort study in France, including patients older than 80 years, Hanon et al. reported significantly lower major bleeding risk in Rivaroxaban-treated patients (7.4/100 patient-years) compared with VKA-treated patients (14.6/100 patient-years) after multivariate adjustment [7]. International guidelines have been published to help clinicians manage anticoagulant-related bleeding [8–10], but their application remains an ongoing challenge [11].

Moreover, most of the studies on anticoagulant-related bleeding have been conducted in emergency rooms and medical departments. A retrospective multicenter study encompassing cases of oral warfarin versus DOACs-associated bleeding events involving individuals older than 66 years, and presenting to five tertiary care hospitals, found that in-hospital mortality was lower following DOAC bleeding events (9.8% vs. 15.2%) [12]. However, there is limited information available regarding the life threatening bleeding events requiring intensive care unit (ICU) admission. This study aimed to describe the epidemiological and clinical characteristics of patients admitted to the ICU for severe extracranial anticoagulant-related bleeding, and to investigate prognosis factors as well as real life therapeutic management.

## Methods

We conducted a multicenter retrospective observational study in five ICUs across different locations in France including University Hospitals in Paris, Strasbourg, Nantes, Lille and Poitiers. The inclusion period spanned 12 years (from January 2007 to December 2018) to collect data before and after DOACs commercialization in France. The data were queried using the ICD-10 nomenclature [13]. We considered all ICD-10 codes indicating potential acute or chronic bleeding diseases and all medical or interventional procedures (Personal list, Additional file 1: Table S1) related to the management of potential bleeding (transfusion, embolization, etc.) for patients receiving therapeutic anticoagulant therapy. Each individual medical record was manually reviewed for patient inclusion. Patients with intracranial bleeding were not included in this study as our focus was to describe severe bleeding responsible for hemorrhagic shock with organ failure. The management and the outcome of intracranial hemorrhage are not dependent on blood volume loss and prognosis factors applicable to intracranial bleeding may not be directly relevant in other cases [14]. General characteristics of patients at admission were collected, including demographic data, biological data, diagnoses, severity of illness evaluated by the Sequential Organ Failure Assessment (SOFA) score [15] and Simplified Acute Physiology Score II (SAPS II) [16]. Additionally, the initial bleeding site and the therapeutic management were recorded. The data are reported in accordance with the STROBE guidelines [17].

### Statistical analysis

Quantitative data are expressed as mean ± standard deviation, qualitative variables as number of recurrences (percentage). Qualitative variables were compared using a Chi2 test. Quantitative variables were compared with a Student's t test or a Mann–Whitney test according to their normal or non-normal distribution (hypothesis of normality systematically tested). The significance threshold was set bilaterally to p < 0.05. Multivariate analysis consisted of a logistic regression including variables available at ICU admission with a p-value < 0.10 in univariate analysis and variables previously identified as predictive for bleeding in patients receiving therapeutic anticoagulant therapy were forced into the model [18], while collinear variables were excluded. As missing data accounted for less than 5% of the dataset, analyses were performed on the complete cases. All statistical analyses were performed using the R software (v 2.12.0; http://cran.rproject.org).

This observational retrospective study had the ethical approval of the SRLF commission (CE SRLF 23-032). All patients and families were informed through the admission leaflet that anonymous data could be used for academic research.

## Results

### Patients’ baseline characteristics

**Fig. 1:**
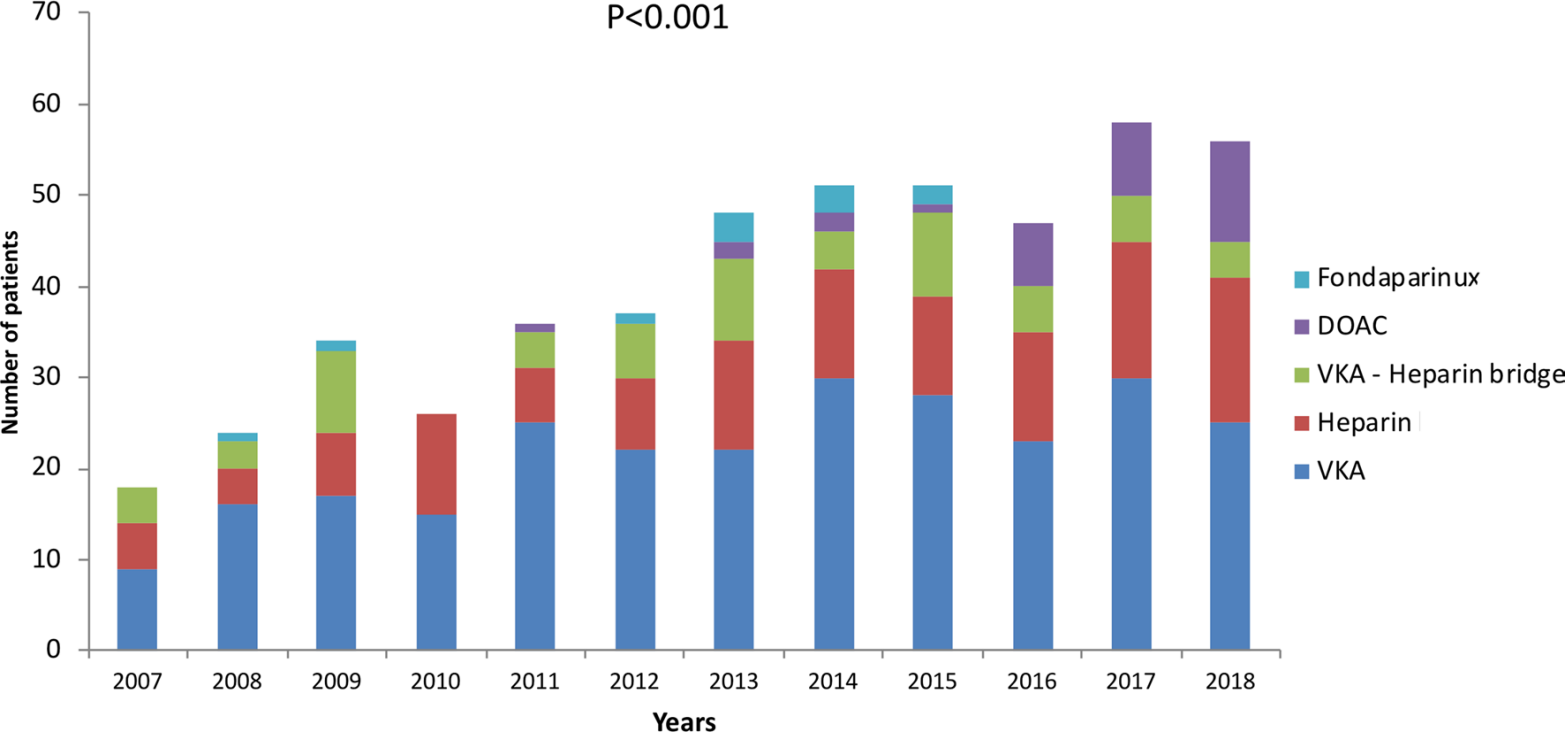
Annual number of patients admitted in ICU for anticoagulant-related extracranial bleeding. *VKA* vitamin K antagonist, *DOAC* direct oral anticoagulant

Among the 95,614 patients admitted to the five ICUs during the inclusion period, 658 patients were admitted for anticoagulant-associated bleeding. After excluding 172 patients with intracranial bleeding, the study cohort comprised 486 patients (N = 161 Strasbourg hospital, N = 117 Saint-Antoine hospital, N = 93 Poitiers hospital, N = 80 Nantes hospital and N = 35 Lille hospital). Approximately half of patients (N = 258, 53%) were admitted to ICU directly from emergency departments. The incidence of extracranial hemorrhagic accidents increased over the study period (Fig. 1), rising from 18 events in 2007 (3.2/1000 admissions) to 56 in 2018 (5.8/1000 admissions). The number of admitted patients treated by heparin or VKA was steady overtime, while the number of DOAC-treated patients gradually increased (Fig. 1).

The patients were predominantly male (61%) with a mean age of 73 ± 13 years, 70% had cardiovascular risk factors and half of them had heart disease (Table 1). 54% of patients were treated by VKA (fluindione, acenocoumarol, warfarin), 25% by heparin (10% unfractionated heparin (UFH), 15% low molecular weight heparin (LMWH)) and 7% received DOACs. Anticoagulants were mostly prescribed for atrial fibrillation (58%) and venous thromboembolic diseases (24%). Patients had several chronic co-medications including antiplatelet agents (33%) and beta-blockers (48%) (Table 1).

**Table 1:** Global characteristics of studied population

Parameters	Value mean (SD) or n (%)
Age, years, mean (SD)	73 ± 13
Male gender	296 (60.9)
*Comorbidities*	
Hypertension	329 (67.7)
Diabetes	160 (32.9)
Cardiopathy	237 (48.8)
Chronic respiratory disease	91 (18.7)
Chronic renal disease	122 (25.1)
Cirrhosis	48 (9.9)
Stroke	76 (15.6)
Hematological malignacies	27 (5.6)
Solid tumor	77 (16.1)
*Associated treatment*	
Antiplatelet therapy	162 (33.3)
Beta blockers	233 (47.9)
*Anticoagulant therapy*	
Vitamin K antagonist	261 (53.7)
Fondaparinux	9 (1.9)
Heparin	119 (24.5)
UFH	49 (10.1)
LMWH	72 (14.8)
VKA—heparin bridge	63 (13.0)
Direct oral anticoagulant	34 (7.0)
Dabigatran	5 (1.0)
Rivaroxaban	17 (3.5)
Apixaban	12 (2.5)
*Anticoagulant therapy indications*	
Deep vein thrombosis	63 (13.0)
Pulmonary embolism	53 (10.9)
Atrial fibrillation	281 (57.8)
Prosthetic valves	62 (12.8)
Others	27 (5.6)

*UFH* unfractionated heparin, *LMWH* low molecular weight heparin, *VKA* vitamin K antagonist

### Organ failure severity

Upon ICU admission, patients exhibited severe organ failure with a high SOFA score (7 ± 4 points) and often required organ support therapy such as vasopressors (31.5%) and invasive mechanical ventilation (34%). Consciousness impairment was frequent (Glasgow score < 15 N = 169, 35%). Regarding biological parameters, we observed a moderate anemia (8.5 ± 2.8 g/dL) and normal platelet counts (228 ± 120 G/L). Hemostasis was impaired as indicated by a prolonged mean aPTT (1.9 ± 1.2 s) and a decreased prothrombin time (PT) (47 ± 24%) (Table 2).

**Table 2:** Clinical and biological characteristics of studied population at admission

Clinical parameters at admission	Mean value (SD) or n (%)
Heart rate, /min	92 ± 23
Mean arterial pressure, mmHg	74 ± 20
Glasgow score	13 ± 4
Urine output, mL/24 h	802 ± 417
*Initial severity scores*	
SOFA	7 ± 4
SAPS II	42 ± 17
*Biologicals*	
Haemoglobin, g/dL	8.5 ± 2.8
Platelet count, G/L	228 ± 120
aPTT ratio, sec	1.9 ± 1.2
Prothrombin ratio	47.0 ± 23.6
Fibrinogen, g/L	3.7 ± 1.7
Creatininemia, µmol/L	169 ± 132
Bilirubin, µmol/L	20 ± 33
Lactate, mmol/L	4.8 ± 5.0
Troponin, µg/L	1.2 ± 8.2
*Organ support therapy in ICU, n (%)*	
Vasopressor	153 (31.5)
Mechanical ventilation	166 (34.2)
*Volume of transfusion*	
Red Blood cells units (n)	3.5 ± 4.0
Platelet units (/10 kg)	4.2 ± 6.0
Fresh Frozen Plasma (mL/kg)	9.2 ± 16.7

*SOFA* Sequential Organ Failure Assessment, *SAPS II* Simplified Acute Physiology Score II, *SD* standard deviation, *aPTT* activated partial thromboplastin time

**Fig. 2:**
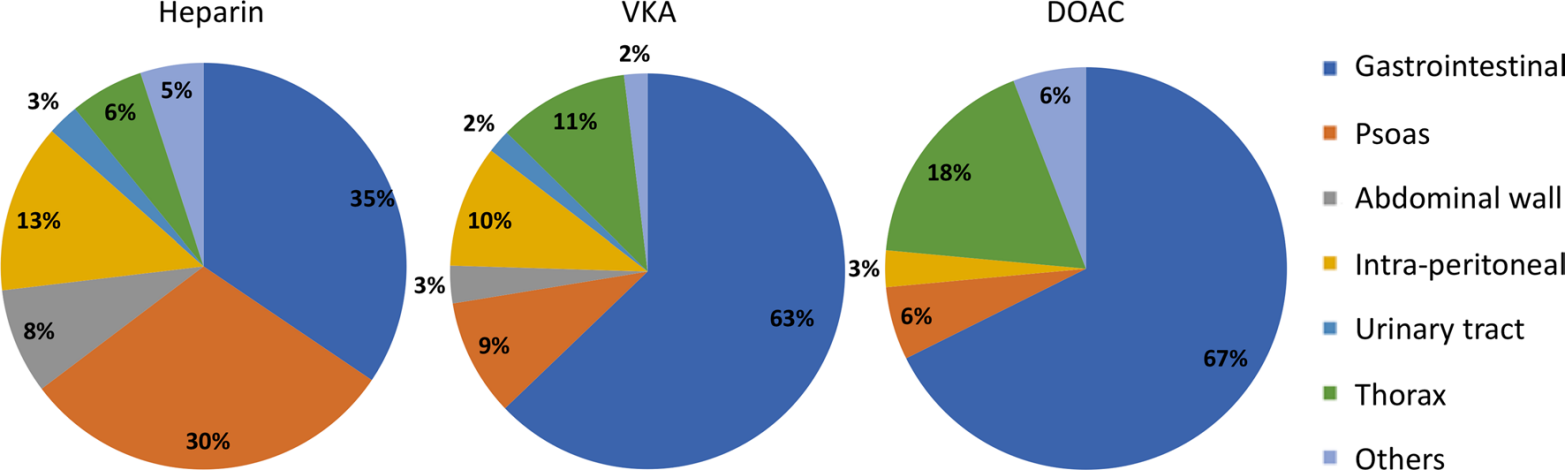
Sites of bleeding depending on type of anticoagulant. *VKA* vitamin K antagonist, *DOAC* direct oral anticoagulant

The primary bleeding sites were the gastrointestinal tract (51%) and abdominal region (psoas 19%, abdominal wall 5%, retroperitoneum 10%). The bleeding sites were substantially different according to type of anticoagulant drug: patients treated with VKA or DOACs primarily experienced bleeding in the gastrointestinal tract, whereas psoas and anterior abdominal wall were the most common bleeding sites in patients receiving heparins (Fig. 2).

### Bleeding management

Upon ICU admission, almost 80% of the patients received red blood cell transfusion. The mean number of packed red blood cells transfused was 3.6 ± 3.3. Additionally, 20% received platelets (mean 0.5 ± 1.3 platelet concentrates) and 39% received fresh frozen plasma (mean 1.7 ± 3.3 units). The management of anticoagulation reversal is detailed in Table 3. For patients treated with VKA, vitamin K was administered in 75% and human prothrombin concentrate (PCC) was used in less than 60%. In the case of heparin treatment, physicians primarily used fresh frozen plasma (50%) and rarely protamine (25%). For DOACs associated bleedings, reversal was performed using frozen plasma (35%), PCC (25%), and activated FVII (7%). The administration of protamine for heparin-treated patients occurred at a median of 3.0 (2.3–3.4) hours after ICU admission, while PCC was administered at 2.6 (2.1–3.2) hours after admission for VKA-treated patients. Among DOACs-treated patients, PCC was injected at a median of 3.1 (2.0–3.9) hours after ICU admission.

**Table 3:** Bleeding management in intensive care units

	VKA n (%)	Heparin n (%)	DOAC n (%)
*Reversal therapy*			
Vitamin K	194 (74.3)	11 (9.2)	2 (5.9)
Protamine	1 (0.4)	26 (21.9)	1 (2.9)
Prothrombin Complex concentrate	157 (60.2)	5 (4.2)	10 (29.4)
Fresh frozen plama	60 (23.0)	58 (48.7)	10 (29.4)
Fibrinogen concentrate	10 (3.8)	5 (4.2)	4 (11.8)
Recombinant factor VIIa	0	0	1 (2.9)
*Intervention for hemostasis control*			
Embolization	24 (11.6)	32 (26.9)	3 (8.8)
Surgery	20 (9.7)	16 (13.5)	2 (5.9)
Endoscopy	103 (49.8)	38 (31.9)	18 (52.9)

The interventional hemostatic management of bleeding varied depending on the bleeding site. Gastrointestinal bleedings were all treated by endoscopic procedures (72.4%) whereas psoas and/or abdominal wall bleedings were mainly treated using endovascular embolization (47.7% and 42.1% respectively) (Additional file 1: Table S3).

### Outcome and prognosis factors

**Fig. 3:**
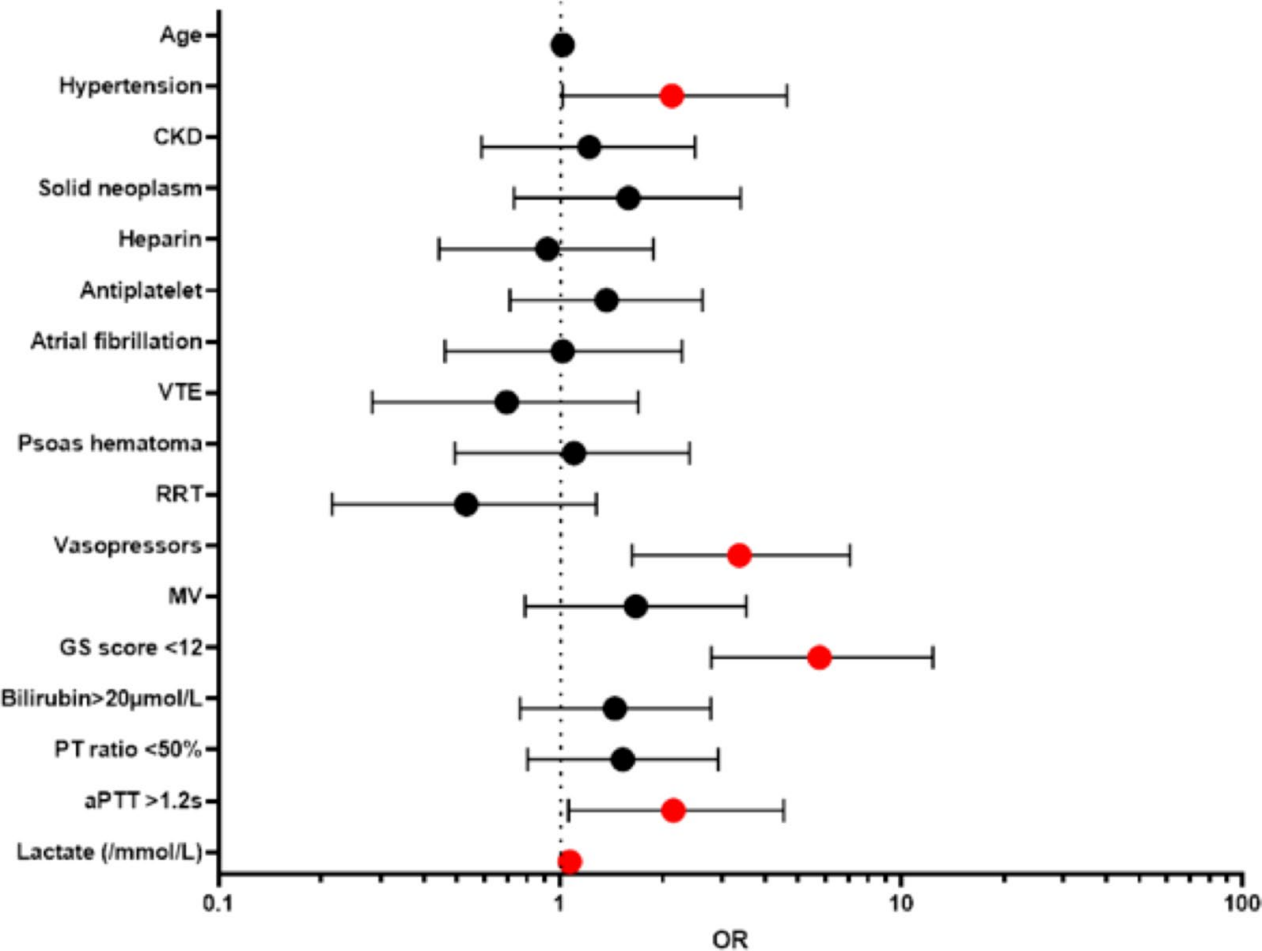
Multivariate analysis of factors associated with ICU mortality. *CKD* chronic kidney disease, *VTE* venous thrombo-embolic disease, *RTT* renal replacement therapy, *MV* mechanical ventilation, *GCS* Glasgow score, *PT* prothrombin time, *aPTT* activated partial prothrombin time, *OR* Odds ratio. Red circle, statistically significant association

ICU mortality was 27% and ICU length of stay was 3 [1–7] days. Univariate analysis revealed that age, hypertension and chronic renal failure were statistically associated with mortality, whereas gender, chronic liver disease and malignancies were not (Additional file 1: Table S2). Non-survivors had a higher SAPS II (56 ± 16 vs. 37 ± 14, p < 0.001), a higher SOFA (10 ± 4 vs. 6 ± 3, p < 0.0001) and more frequently received organ support therapy such as mechanical ventilation (90% vs. 76%, P < 0.001) and vasopressors (86% vs. 67%, P < 0.001). Additionally, non-survivors had lower Glasgow Coma Scale scores upon admission (10 ± 5 vs. 14 ± 3, p < 0.0001) and showed more pronounced coagulation impairment and kidney injury. The incidence of anticoagulant overdose did not differ between survivors and non survivors (p = 0.53). However, non-survivors received more FFP and platelets (p < 0.0001 and p = 0.003 respectively) with no significant difference in the number of RBC units transfused. Finally, neither the type of anticoagulant, the bleeding site nor the rate of concomitant anti-platelet medication were statistically different between survivors and non-survivors. In the multivariate analysis, five factors were found to be independently associated with mortality: hypertension, need for vasopressors, impaired consciousness, hyperlactatemia and prolonged aPTT (Fig. 3; Additional file 1: Table S4).

## Discussion

This multicenter retrospective study showed that over the past decade the number of patients admitted to ICU for anticoagulant-related extracranial bleeding has significantly increased. These patients experienced severe organ failure, requiring frequent organ support therapy and ultimately faced a high mortality rate. One concerning finding of our study is the weak compliance with guidelines for managing anticoagulant-related bleeding. Enhancing adherence to these guidelines could be crucial to improve patient outcomes.

In our cohort, critically ill patients admitted to ICU for anticoagulant-related extracranial bleeding accounted for around 1% of ICU patients, consistent with previous works [2, 19]. The increase in ICU admission for severe bleeding complications can be imputed to the growing use of anticoagulants in France [2] driven by the aging population and broader indications for anticoagulants, especially for atrial fibrillation which accounted for 60% of anticoagulant prescriptions in our cohort [20]. DOAC-related bleedings were identified starting from 2012, 2 years after their introduction to the market. The continuous increase of DOAC prescription suggests that physicians should receive specific training to effectively manage bleeding occurring with dabigatran, rivaroxaban or apixaban-therapy. Previous studies have shown that among DOACs, dabigatran was at higher risk of bleeding events [6, 21, 22]. In our study dabigatran represented only 1% of anticoagulant related bleeding accidents. We also observed an encouraging decrease in the number of patients admitted with bleeding occurring during VKA-heparin bridge in recent years. This could be explained by the implementation of recent expert international recommendations that propose limiting VKA-heparin bridge for elective surgery [23].

Consistently with the work of Hauguel et al. [24] patients were elderly, with a high prevalence of cardiovascular risk factors and comorbidities. In our cohort, hypertension was not found to be independently associated with ICU mortality. This contrasts with the findings of Goodman et al. who observed a significant relationship between hypertension and the outcome in bleeding among VKA- and rivaroxaban-treated patients [25]. However, Lauzier et al., who focused on heparin-treated patients, did not report such an association [26]. Here, one third of patients were treated by antiplatelet medication in addition to anticoagulants, but this combination was not associated with in-ICU mortality. Similar results were reported by Beyer-Westendorf et al. [27] in selected rivaroxaban-treated patients that frequently received antiplatelet agents (16%), with no association with bleeding severity.

Bleeding sites were different according to type of anticoagulant drug. In our cohort and in other studies [21–27], VKA or DOACs-treated patients predominantly experienced bleeding in the digestive tract. The mechanisms underlying such observations is unclear. Some authors have speculated that the preferential localization of bleeding in the digestive tract with DOACs may be related to their activity on the P-glycoprotein transporter [28]. In contrast heparin-treated patients presented with bleeding in soft tissues such as the psoas muscle or the abdominal wall. In the multicenter study by Litjos et al. [29] which examined psoas hematomas managed in the ICU, almost half of the patients were treated by heparin. For abdominal anterior wall hematomas, Hauguel et al. described iatrogenic and traumatic mechanisms due to the puncture of hypogastric artery branches during subcutaneous heparin injection [24].

Upon admission, patients in our study exhibited high severity, frequently presenting with organ failure and requiring both vasopressors and invasive mechanical ventilation. These findings are consistent with the monocentric study by Hauguel et al. [24]. The overall in-ICU mortality from anticoagulant-related extracranial hemorrhagic accidents admitted to ICU was 27% in our cohort, which aligns with the all-cause mortality in French ICUs (23%) [30].

Factors found to be associated with mortality in our cohort are very difficult to compare to published works because the definition of severe bleeding varies from one study to another [31]. Here, these factors reflected the severity of acute circulatory failure (vasopressor need, hyperlactatemia, and impaired consciousness). Furthermore, prolonged aPTT was also independently associated with mortality. Whether this is due to the anticoagulant drug or to hemorrhage-associated liver failure or consumption coagulopathy is uncertain.

Finally, one of the main findings of this work is the lack of compliance with expert recommendations on anticoagulation reversal [8–10]. For instance, only 60% of patients with severe VKA-associated hemorrhagic accidents received PCC, and only 22% of patients admitted for heparin-associated bleeding received protamine. In the emergency ward, Tremey et al. also reported inappropriate use of PCC in these patients, which improved after a teaching program [11]. Starting from 2012 bleeding accidents associated with DOACs became common in ICU. Before Idarucizumab and Andexanet alfa became available, PCC administration was recommended. In our cohort, PCCs were only used in 29% of cases and fresh frozen plasma was used in 29% of cases. These results underscores the need for developing of teaching programs for anticoagulant-related bleeding management in ICUs.

We did not find any significant relationship between prognosis and time to reversal and/or hemostatic procedures but such an association cannot be excluded. Indeed the retrospective nature of our work is a limitation and despite the exhaustive analysis of each medical record, we cannot rule out the possibility of missing data, particularly regarding reversal therapy before ICU admission. In addition, the ICD-10 code-based approach used in this study to identify the cases is original and not validated by previous studies. Finally, our study included only 5 medical ICUs, thereby limiting the generalizability of its results, particularly for post-operative patients. Therefore, future prospective research should be conducted to validate our observations.

## Conclusion

In this retrospective multicenter study, we identified a concerning rise in ICU admission for severe extracranial anticoagulant-related bleeding. The severity of these events was notably high, and occurred in elderly patients with cardiovascular comorbidities, resulting in a significant in-ICU mortality rate. Their management currently seems suboptimal, highlighting the urgent need for improvement through teaching programs.

### Supplementary Information

**Additional file 1. Table S1: **Personal list for the screening of patients admitted in ICU for anticoagulant-related bleeding, based on ICD-10 nomenclature.

## Data Availability

The data sets used and analyzed during the current study are available from the corresponding author upon reasonable request.

## Abbreviations

DOAC: Direct oral anticoagulant
VKA: Vitamin K antagonist
ICU: Intensive care unit
SRLF: Société de Réanimation de Langue Française
FFP: Fresh frozen plasma
SOFA: Sequential Organ Failure Assessment
