# Supplementary material for: Extracranial anticoagulant related bleedings admitted to intensive care units: a French multicenter retrospective study
Source: Crit Care. 2023 Aug 9;27:312. doi: 10.1186/s13054-023-04605-4 (PMC10411017; doi:10.1186/s13054-023-04605-4)
Supplement: Supplementary file 1 — Additional file 1. Table S1: Personal list for the screening of patients admitted in ICU for anticoagulant-related bleeding, based on ICD-10 nomenclature. [file 13054_2023_4605_MOESM1_ESM.docx]

| ICD10 | ICD10 LABEL | ICD10 | ICD10 LABEL | ICD10 | ICD10 LABEL |
| --- | --- | --- | --- | --- | --- |
| D500 | Iron deficiency anaemia secondary to blood loss (chronic) | I779 | Disorder of arteries and arterioles, unspecified | N950 | Menopausal and other perimenopausal disorders |
| D508 | Other iron deficiency anaemias | I78 | Diseases of capillaries | R04 | Haemorrhage from respiratory passages |
| D509 | Iron deficiency anaemia, unspecified | I841 | Internal haemorrhoids with other complications | R233 | Spontaneous ecchymoses |
| D62 | Acute posthaemorrhagic anaemia | I844 | External haemorrhoids with other complications | R31 | Unspecified haematuria |
| D683 | Haemorrhagic disorder due to circulating anticoagulants | I850 | Oesophageal varices | R571 | Hypovolaemic shock |
| D684 | Acquired coagulation factor deficiency | I87 | Other disorders of veins | S064 | Epidural haemorrhage |
| D688 | Other specified coagulation defects | I97 | Postprocedural disorders of circulatory system, not elsewhere classified | S065 | Traumatic subdural haemorrhage |
| D689 | Coagulation defect, unspecified | I9820 | Oesophageal varices in diseases classified elsewhere | S066 | Traumatic subarachnoid haemorrhage |
| D69 | Purpura and other haemorrhagic conditions | J942 | Haemothorax | S202 | Contusion of thorax |
| G46 | Vascular syndromes of brain in cerebrovascular diseases | K226 | Gastro-oesophageal laceration-haemorrhage syndrome | S271 | Traumatic haemothorax |
| G951 | Vascular myelopathies | K25 | Gastric ulcer | S272 | Traumatic haemopneumothorax |
| H113 | Conjunctival haemorrhage | K26 | Duodenal ulcer | S300 | Contusion of lower back and pelvis |
| H114 | Other conjunctival vascular disorders and cysts | K27 | Peptic ulcer, site unspecified | S301 | Contusion of abdominal wall |
| H210 | Hyphaema | K28 | Gastrojejunal ulcer | S400 | Contusion of shoulder and upper arm |
| H211 | Other vascular disorders of iris and ciliary body | K290 | Gastritis and duodenitis | S500 | Superficial injury of forearm |
| H313 | Choroidal haemorrhage and rupture | K518 | Other ulcerative colitis | S501 | Contusion of other and unspecified parts of forearm |
| H356 | Retinal haemorrhage | K519 | Ulcerative colitis, unspecified | S700 | Contusion of hip |
| H431 | Vitreous haemorrhage | K625 | Haemorrhage of anus and rectum | S701 | Contusion of thigh |
| H450 | Vitreous haemorrhage in diseases classified elsewhere | K661 | Haemoperitoneum | S800 | Contusion of knee |
| H922 | Otorrhagia | K762 | Central haemorrhagic necrosis of liver | S801 | Contusion of other and unspecified parts of lower leg |
| I230 | Haemopericardium as current complication following acute myocardial infarction | K920 | Haematemesis | T45 | Poisoning by anticoagulants |
| I312 | Haemopericardium, not elsewhere classified | K921 | Melaena | T46 | Poisoning by agents primarily affecting the cardiovascular system |
| I60 | Subarachnoid haemorrhage | K922 | Gastrointestinal haemorrhage, unspecified | T792 | Traumatic secondary and recurrent haemorrhage |
| I61 | Intracerebral haemorrhage | M250 | Haemarthrosis | T810 | Haemorrhage and haematoma complicating a procedure, not elsewhere classified |
| I62 | Other nontraumatic intracranial haemorrhage | N421 | Congestion and haemorrhage of prostate | T811 | Shock during or resulting from a procedure, not elsewhere classified |
| I64 | Stroke, not specified as haemorrhage or infarction | N836 | Haematosalpinx | X44 | Accidental poisoning by and exposure to other and unspecified drugs, medicaments and biological substances |
| I679 | Cerebrovascular disease, unspecified | N837 | Haematoma of broad ligament | X64 | Intentional self-poisoning by and exposure to other and unspecified drugs, medicaments and biological substances |
| I68 | Cerebrovascular disorders in diseases classified elsewhere | N857 | Haematometra | Y14 | Poisoning by and exposure to other and unspecified drugs, medicaments and biological substances, undetermined intent |
| I69 | Sequelae of cerebrovascular disease | N897 | Haematocolpos | Y44 | Agents primarily affecting blood constituents |
| I772 | Rupture of artery | N92 | Excessive, frequent and irregular menstruation | Z921 | Personal history of long-term (current) use of anticoagulants |
| I778 | Other specified disorders of arteries and arterioles | N93 | Other abnormal uterine and vaginal bleeding | Z5130 | Blood transfusion without reported diagnosis |

| CCAM | CCAM LABEL | CCAM | CCAM LABEL | CCAM | CCAM LABEL |
| --- | --- | --- | --- | --- | --- |
| DDSF001 | Embolization or closure of a coronary fistula or aneurysm | EDSF014 | Selective and/or hyperselective embolization of several branches of the internal iliac artery or of several extra-digestive branches of the abdominal aortaroute | FELF006 | Transfusion of non-erythrocyte labile blood product |
| DFSF002 | Embolization of intrathoracic arteriovenous fistula | EDSF015 | Supraselective embolization of several digestive arteries | FELF008 | Transfusion of packed red blood cells, at home |
| EASF004 | Supraselective unilateral or bilateral branch embolization of the internal carotid artery | EDSF016 | Selective or hyperselective embolization of the internal iliac artery or an extra-digestive branch of the abdominal aorta | FELF011 | Transfusion of packed red blood cells with a volume of less than half the blood mass |
| EASF005 | Transcutaneous arterial and venous embolization of a multipedicular cranioencephalic dural arteriovenous fistula | EESF004 | Hyperselective embolization of several arteries of the lower limb | GASE001 | Intranasal hemostasis by cauterization or electrocoagulation |
| EASF006 | Embolization of a unipedicular cranioencephalic dural arteriovenous fistula | EESF006 | Supraselective embolization of an artery of the lower limb | GASE002 | Intranasal hemostasis by unilateral or bilateral transmucosal injection of pharmacological agent |
| EASF009 | Transcutaneous arterial and venous embolization of a unipedicular cranioencephalic dural arteriovenous fistula | EESF007 | Selective or hyperselective embolization of an artery of the lower limb | GELE007 | Intrabronchial placement of haemostatic material, by rigid tube bronchoscopy |
| EASF014 | Selective or hyperselective unilateral or bilateral embolization of the branch of the internal carotid artery | EGSF001 | Supraselective embolization of the testicular or ovarian vein | HASD003 | Hemostasis secondary to intraoral electrocautery |
| EASF015 | Arterial or transcutaneous venous embolization of a multipedicular cranioencephalic dural arteriovenous fistula | EGSF002 | Selective or hyperselective embolization of the testicular or ovarian vein | HBSD001 | Gingivoalveolar hemostasis secondary to dental avulsion |
| EBSF003 | Selective or hyperselective unilateral or bilateral embolization of the branch of the external carotid artery | EGSF003 | Embolization of the draining veins of the penis | HESE001 | Hemostasis of lesion of the esophagus, stomach and/or duodenum with laser |
| EBSF004 | Supraselective unilateral or bilateral embolization of the branch of the external carotid artery | EHBD001 | Packing of oesogastric varices by balloon catheter | HESE002 | Hemostasis of lesion of the esophagus, stomach and/or duodenum without laser |
| ECSF002 | Supraselective embolization of an artery of the upper limb, by transcutaneous arterial route | EHNE001 | Session of sclerosis and/or ligation of oesogastric varices outside the bleeding period | HGSE001 | Hemostasis of intestinal lesion without laser |
| ECSF004 | Selective or hyperselective artery embolization for bronchial or pleuropulmonary destination | EHNE002 | Sclerosis and/or ligation of oesogastric varices during bleeding | HGSE002 | Hemostasis of intestinal lesion with laser |
| ECSF006 | Supraselective artery embolization for bronchial or pleuropulmonary destination | EHSF001 | Embolization of oesogastric varices or intrahepatic branches of the portal vein | HGSE003 | Hemostasis of intestinal lesion without laser |
| ECSF008 | Selective or hyperselective embolization of an artery of the upper limb | EJFB001 | Excision session of a segment of varicose vein or perforating vein of the lower limb | HGSE004 | Hemostasis of intestinal lesion with laser |
| EDLF004 | Placement of a stent graft in the common iliac artery and/or the external iliac artery with embolization of the internal iliac artery | ELSF001 | Embolization of a renal arteriovenous fistula | HHSE001 | Hemostasis of colon lesion with laser |
| EDSF003 | Selective or hyperselective embolization of the renal artery | ENSF001 | Embolization of a vascular malformation or vertebral lesion | HHSE002 | Hemostasis of colon lesion without laser |
| EDSF004 | Supraselective embolization of the branch of the internal iliac artery or of the extra-digestive branch of the abdominal aorta | ENSF002 | Embolization of intraparenchymal arteriovenous malformation of the spinal cord | HHSE003 | Hemostasis of colon lesion with laser |
| EDSF005 | Selective or hyperselective embolization of a digestive artery | ENSF003 | Transcutaneous vascular embolization of spinal dural arteriovenous malformation | HHSE004 | Hemostasis of colon lesion without laser |
| EDSF006 | Supraselective embolization of a digestive artery | EZSF001 | Definitive intraoperative occlusion of a vascular trunk by balloon or embolization | HJSD001 | Hemostasis secondary to an act on the rectum |
| EDSF008 | Supraselective embolization of the renal artery | FELF001 | Transfusion of packed red blood cells with a volume greater than half a blood mass, during an operation under general or locoregional anesthesia | HKSD001 | Hemostasis secondary to an act on the anus |
| EDSF011 | Transcutaneous arterial embolization of the internal [hypogastric] iliac arteries and/or its branches for postpartum hemorrhage | FELF003 | Simultaneous intravenous administration of two of the following blood products: fresh frozen plasma, platelet | LBLD003 | Placement of a maxillary or mandibular gutter for hemostasis or topical carrier |
| EDSF012 | Selective and/or hyperselective embolization of several digestive arteries | FELF004 | Transfusion of packed red blood cells with a volume greater than half a blood mass in adults | LBLD006 | Placement of maxillary and mandibular splints for hemostasis or topical carrier |

**Supplemental table 1 A-B : list of ICD codes for patients’ screening**

| **Variables** | Alive  Mean (SD) | Died  Mean (SD) | p |
| --- | --- | --- | --- |
| **Age, years** | 72 ± 14 | 75 ± 10 | **0.024** |
| **Female gender, n (%)** | 142 (39.9) | 48 (36.9) | 0.553 |
| **Arterial hypertension** | 230 (64.6) | 99 (76.2) | **0.018** |
| **Diabetes** | 112 (31.5) | 48 (36.9) | 0.257 |
| **Chronic heart disease** | 188 (52.8) | 77 (59.2) | 0.208 |
| **Chronic respiratory failure** | 65 (18.3) | 26 (20.0) | 0.885 |
| **Chronic renal failure** | 81 (22.8) | 41 (31.5) | **0.048** |
| **Chronic liver failure** | 34 (9.6) | 14 (10.8) | 0.690 |
| **Stroke** | 51 (14.3) | 25 (19.2) | 0.188 |
| **Obliterating arteriopathy** | 47 (13.2) | 17 (13.1) | 0.975 |
| **Hematological malignancy** | 20 (5.6) | 7 (5.4) | 0.921 |
| **Solid tumor** | 51 (14.3) | 27 (20.8) | 0.087 |
| **Antiplatelet therapy** | 123 (34.6) | 39 (30.0) | 0.346 |
| **Beta blockers** | 163 (45.8) | 70 (53.9) | 0.122 |
| **VKA** | 196 (55.1) | 65 (50.0) | 0.322 |
| **Heparin** | 79 (22.2) | 41 (31.5) | 0.052 |
| **UFH** | 33 (9.3) | 16 (12.3) | 0.325 |
| **LMWH** | 47 (13.2) | 25 (19.2) | 0.098 |
| **VKA - heparin bridge** | 47 (13.2) | 16 (12.3) | 0.795 |
| **Fondaparinux** | 6 (1.7) | 3 (2.3) | 0.652 |
| **DOAC** | 29 (8.2) | 5 (3.9) | 0.132 |
| **Dabigatran** | 4 (1.1) | 1 (0.8) | 0.732 |
| **Rivaroxaban** | 15 (4.2) | 2 (1.5) | 0.155 |
| **Apixaban** | 10 (2.8) | 2 (1.5) | 0.424 |
| **Venous thrombo-embolic diseases** | 96 (27.0) | 17 (14.7) | **0.008** |
| **Deep vein thrombosis** | 55 (15.5) | 8 (6.9) | **0.007** |
| **Pulmonary embolism** | 41 (11.5) | 9 (7.8) | 0.474 |
| **Atrial fibrillation** | 196 (55.1) | 77 (66.4) | **0.041** |
| **Prosthetic heart valves** | 46 (12.9) | 15 (12.9) | 0.858 |
| **Others** | 5 (1.4) | 7 (6.0) | 0.497 |
| **Gastrointestinal** | 181 (50.9) | 66 (50.8) | 0.989 |
| **Psoas** | 56 (15.7) | 30 (23.1) | 0.060 |
| **Anterior abdominal wall** | 20 (5.6) | 7 (5.4) | 0.921 |
| **Intra/Retroperitoneal** | 45 (12.6) | 13 (10.0) | 0.427 |
| **Hematuria** | 5 (1.4) | 3 (2.3) | 0.489 |
| **Hemoptysis / Hemothorax** | 37 (10.4) | 10 (7.7) | 0.373 |
| **At admission** | | | |
| **Heart rate, /min** | 91 ± 21 | 95 ± 26 | 0.373 |
| **Mean arterial pressure, mmHg** | 76 ± 11 | 61 ± 20 | **< 0.001** |
| **Glasgow score** | 14 ± 3 | 10 ± 5 | **< 0.001** |
| **Urine output, mL/24h** | 1113 ± 447 | 689 ± 342 | **< 0.001** |
| **Vasopressor** | 86 (24.2) | 67 (51.5) | **< 0.001** |
| **Mechanical ventilation** | 76 (21.4) | 90 (69.2) | **< 0.001** |
| **SOFA** | 6 ± 3 | 10 ± 4 | **< 0.001** |
| **SAPS II** | 37 ± 14 | 56 ± 16 | **< 0.001** |
| **Haemoglobin, g/dL** | 8.5 ± 2.7 | 8.6 ± 3.1 | 0.739 |
| **Platelet count, G/L** | 231.4 ± 119.4 | 218.8 ± 120.3 | 0.202 |
| **aPTT, sec** | 1.8 ± 1.2 | 2.0 ± 1.3 | **< 0.001** |
| **Prothrombin ratio** | 48.8 ± 23.6 | 42.0 ± 23.0 | **0.005** |
| **Fibrinogen, g/L** | 3.8 ± 1.6 | 3.5 ± 1.7 | 0.117 |
| **Creatininemia,** µ**mol/L** | 160.3 ± 134.0 | 194.1 ± 123.4 | **< 0.001** |
| **Bilirubin,** µ**mol/L** | 17.8 ± 19.7 | 26.4 ± 53.2 | **0.009** |
| **Lactate, mmol/L** | 3.6 ± 3.9 | 7.5 ± 6.2 | **< 0.001** |
| **Troponin,** µ**g/L** | 0.8 ± 5.3 | 1.8 ± 10.2 | 0.081 |
| **Time ICU admission -> medical management, n (%)** | Alive | Died | p |
| **D < 6h** | 152 (75.6) | 65 (81.3) | 0.577 |
| **6h < D < 12h** | 8 (4.0) | 2 (2.5) |  |
| **D > 12h** | 41 (20.4) | 13 (16.3) |  |
| **Time ICU admission -> intervention, n (%)** |  |  |  |
| **D < 6h** | 112 (44.6) | 43 (53.1) | 0.366 |
| **6h < D < 12h** | 54 (21.5) | 13 (16.1) |  |
| **D > 12h** | 85 (33.9) | 25 (30.9) |  |
| **Medical management, n (%)** | | | |
| **Fresh frozen plasma** | 105 (29.5) | 52 (40.0) | 0.123 |
| **Vit K / VKA** | 146 (74.5) | 48 (73.9) | 0.591 |
| **PCC / VKA** | 121 (61.7) | 36 (55.4) | 0.342 |
| **Vit K + PCC / VKA** | 99 (50.5) | 31 (47.7) | 0.503 |
| **Protamine / Heparin** | 17 (21.5) | 9 (22.0) | 0.380 |
| **PCC / DOAC** | 10 (34.5) | 0 (0.0) | 0.057 |
| **Red blood cells, units** | 3 ± 3 | 4 ± 4 | 0.461 |
| **Platelets, (units/10kg)** | 2,4 ± 6.6 | 4.8 ± 7.2 | **0.003** |
| **Frozen plasma (mL/Kg)** | 5.0 ± 9.6 | 12.1 ± 20.8 | **< 0.001** |

**Supplemental Table 2 : comparison of patients’ characteristics according to ICU outcome.** aPTT, activated partial thromboplastin time; CKD, chronic kidney disease; DOAC, direct oral anticoagulant; GCS, Glasgow score; VKA, vitamin K antagonist; SAPS II, Simplified Acute Physiology Score II; SOFA, Sequential Organ Failure Assessment;

| **Hemostatic**  **treatment** | Gastrointestinal  n (%) | Abdominal  Psoas  n (%) | Abdominal  Non psoas  n (%) | Urinary tract  n (%) | Lung  Thorax  n (%) |
| --- | --- | --- | --- | --- | --- |
| Embolization | 10 (4.1) | 41 (47.7) | 32 (42.1) | 1 (12.5) | 14 (29.8) |
| Surgery | 13 (5.3) | 11 (12.8) | 12 (15.8) | 1 (12.5) | 6 (12.8) |
| Endoscopy | 176 (72.4) | 2 (2.3) | 4 (5.3) | 1 (12.5) | 9 (19.2) |

**Supplemental table 3: hemostatic procedures according to the bleeding site.**

|  | OR | Lower 95% CI | Upper 95% CI | *P* value |
| --- | --- | --- | --- | --- |
| Age (/year)^a,b^ | 1.02 | 0.985 | 1.05 | 0.330 |
| Hypertension^a,b^ | 2.13 | 1.02 | 4.63 | 0.048 |
| CKD^a,b^ | 1.22 | 0.59 | 2.49 | 0.586 |
| Solid neoplasm^a^ | 1.59 | 0.734 | 3.39 | 0.235 |
| Heparin^a^ | 0.919 | 0.443 | 1.88 | 0.817 |
| Anti-platelet^a,b^ | 1.37 | 0.714 | 2.62 | 0.343 |
| Atrial fibrillation^a^ | 1.02 | 0.461 | 2.28 | 0.962 |
| VTE^a^ | 0.699 | 0.282 | 1.7 | 0.431 |
| Psoas bleeding^a^ | 1.1 | 0.493 | 2.4 | 0.816 |
| RRT^a^ | 0.532 | 0.215 | 1.28 | 0.162 |
| Vasopressors^a^ | 3.36 | 1.63 | 7.08 | 0.001 |
| MV^a^ | 1.67 | 0.791 | 3.52 | 0.175 |
| GS score <12^a^ | 5.77 | 2.78 | 12.4 | <0.0001 |
| Bilirubin >20µmol/L^a^ | 1.45 | 0.765 | 2.77 | 0.252 |
| PT ratio <50%^a^ | 1.53 | 0.805 | 2.91 | 0.195 |
| aPTT >1.2s^a^ | 2.15 | 1.06 | 4.53 | 0.038 |
| Lactate (/mmol/L) ^a^ | 1.07 | 1.01 | 1.14 | 0.032 |

**Supplemental table 4. Multivariate analysis for factors associated with ICU mortality.** a, variables with P value <0.10 in the univariate analysis ;b, variables identified as associated with outcome in previous works (see reference 18). CKD, chronic kidney disease; GS, Glasgow score; VTE, venous thrombo-embolic disease; RRT, renal replacement therapy; MV, mechanical ventilation; PT, prothrombin time; aPTT, activated partial thromboplastin time.
